# Molecular genetic and biochemical characterization of a putative family of zinc metalloproteins in *Caenorhabditis elegans*†
†Electronic supplementary information (ESI) available. According to the UK Research Councils' Common Principles on Data Policy, all data supporting this study are openly available. See DOI: 10.1039/c8mt00169c


**DOI:** 10.1039/c8mt00169c

**Published:** 2018-11-16

**Authors:** Poulami Chaudhuri, Hasan Tanvir Imam, Yona Essig, Jovaras Krasauskas, Samuel M. Webb, Claudia A. Blindauer, Stephen R. Stürzenbaum

**Affiliations:** a King's College London , School of Population Health & Environmental Sciences , Faculty of Life Sciences & Medicine , 150 Stamford Street , London , SE1 9NH , UK . Email: stephen.sturzenbaum@kcl.ac.uk; b University of Warwick , Department of Chemistry , Gibbet Hill , Coventry , CV4 7AL , UK; c Stanford Synchrotron Radiation Lightsource , SLAC National Accelerator Laboratory , 2575 Sand Hill Road , Menlo Park , CA 94025 , USA

## Abstract

Four highly similar genes (*W08E12.2*, *W08E12.3*, *W08E12.4* and *W08E12.5)* which are consecutively aligned on chromosome IV of the *C. elegans* genome are predicted to code for small (120–141aa) yet cysteine rich (18–19 cysteines) proteins. Cloning and sequencing of the genomic regions of the isoforms confirmed the presence and order of all genes. The generation of transgenic worms strains with an integrated single copy or extrachromosomal multi-copy P*W08E12.3;W08E12.4*::GFP uncovered that *W08E12.3* and *W08E12.4* are constitutively expressed in the pharynx and significantly induced in worms exposed to 100 μM Zn. Knockdown by RNAi did not have a marked consequence on reproductive performance nor was a Zn-dependent effect on nematode growth observed. However, RNAi of these genes led to an accumulation of Zn in the intestinal cells. W08E12.3 was recombinantly expressed in *E. coli* and the purified protein was shown to be able to bind up to 6.5 Zn molecules at neutral pH. Zn-binding was acid-labile and the apo protein was observed at pH < 4.3. This characterization suggests *W08E12.2*, *W08E12.3*, *W08E12.4* and *W08E12.5* belong to a family of putative Metalloproteins which, akin to metallothioneins, may play an important role in Zn-sensing, homeostasis and/or detoxification.

## Abstract 2

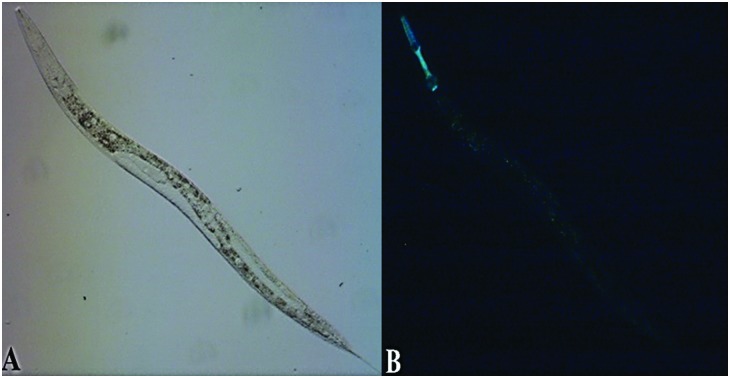
The first characterization of *W08E12.2*, *W08E12.3*, *W08E12.4* and *W08E12.5*, four putative metalloproteins in *C. elegans*. (A) phase contrast microscopy, (B) fluorescence microscopy of *PW08E12.3*;*W08E12.4*::GFP.

##

Significance to metallomics

*C. elegans* metallothionein and phytochelatin synthase mutants are surprisingly resilient when challenged with a toxic load of heavy metals. This suggests that other, yet to be identified, metalloproteins may also be involved in essential metal homeostasis and non-essential metal detoxification. This paper provides the first characterization of a putative family of metalloproteins, focusing primarily on molecular genetic and biochemical studies to define their involvement in Zn metabolism/detoxification.

## 1. Introduction

*C. elegans* is an ideal model to study toxicity and toxicological mechanisms of heavy metals.^1^ Most studies have focused on toxic endpoints including reproduction, life span, lethality and protein expression. More specifically, the effect of metals on the nervous system has been studied by investigating the behaviour, reporter expression and neuronal morphology.^2^ Moreover, silencing methodologies such as knockdown by RNAi or the generation of chromosomal deletions provide opportunities for a targeted manipulation of specific genes at the molecular level. This has helped to unveil information about how certain mutants are more sensitive to metal toxicity.^3^–^5^ The transparent body of *C. elegans* allows the visualization of fluorescently labelled molecules, genes and proteins *in vivo*,^6^ a feature that can aid in the investigation of metal detoxification pathways and is amenable for assessing the interaction between the genes and environmental factors. The role of the nematode as a biosensor for environmental risk assessment has been explored by exploiting the current knowledge of established biomarkers such as glutathione (GSH), metallothionein (MT), heat shock proteins (HSPs) and pumps and transporters involved in metal detoxification. *C. elegans* is thus an indispensable model to complement more classical (mammalian) toxicological research.^2^,^7^

The homeostatic control of heavy metals is biologically complex as it requires an intricate balance between maintaining essential micronutrients (*e.g.* Zn and Cu) and the detoxification of harmful metals (*e.g.* Cd). Two prominent pathways involved in this challenge are the phytochelatins (PCs)/phytochelatin synthase (*pcs-1*)^8^–^11^ and the MTs (in *C. elegans* nomenclature MTs are referred to as MTLs).^12^–^16^ PCs are a family of metal-inducible thiol-rich peptides that are synthesised enzymatically and play a prominent role in the detoxification of heavy metals by acting as chelators.^17^ The PCs form complexes with toxic metals in the cytosol of the cell which are transported into the vacuole, protecting the organism from heavy metal toxicosis^18^ and the biosynthesis of PCs is auto-regulated by this metal chelation.^19^ Unlike PCs, expression of metallothionein is driven by transcriptional activation and the resultant proteins bind metals and act as antioxidants.^20^*C. elegans* possesses two metallothionein (MT) genes, *mtl-1* and *mtl-2*, which have been shown to play an important role in the protection from metal toxicity. They are thought to have distinct functions. The *mtl-1* is constitutively expressed in the lower pharyngeal bulb in the absence of metal exposure and thus may act as a metal sensor. The expression of *mtl-1* and *mtl-2* is elevated in the gut region upon metal exposure. Both isoforms have preferences for metal binding, where *mtl-1* is biased towards Zn(ii) and *mtl-2* towards Cd(ii).^16^,^21^ The single and double knockout of *mtl-1* and/or *mtl-2* as well as the *mtl-1;mtl-2;pcs-1* triple knockout mutants are all characterized by an increased sensitivity (compared to wild type) when challenged with high doses of Zn or Cd, at least in terms of development and reproduction.^10^,^22^ However, given that the mutants are all viable and capable of producing offspring suggests that other, hitherto uncharacterized metalloproteins must play equally important roles in essential metal homeostasis and non-essential metal detoxification.

The *C. elegans* whole genome sequencing effort^23^ has allowed the building of WormBase (; www.wormbase.org), an exquisitely detailed database. A manual screen of the database identified four highly conserved genes that are chronologically arranged on chromosome IV. Annotated as *W08E12.2*, *W08E12.3*, *W08E12.4*, and *W08E12.5* (based on their position within the sequenced cosmid W08E12) they are predicted to encode cysteine-rich proteins. The sequences are highly similar to each other as well as to uncharacterized genes present in other *Caenorhabditis* species (; www.wormbase.org). Given that the *C. elegans* genome is compact (with just over 100 million bp encoding for about 20 000 proteins), multi-copy isoforms/isomers are less frequent compared to higher organisms with more complex genomes. The aligned (sequential) occurrence of numerous members of a highly similar gene family within a compact genome is therefore relatively rare and highlights that their role within the nematode is likely to be significant. Moreover, the abundance and conservation of cysteine residues within *W08E12.2*, *W08E12.3*, *W08E12.4* and *W08E12.5* suggests that this family may be involved in the binding of heavy metals, a notion this paper sets out to explore in more detail.

## 2. Materials and methods

### 2.1 Nematode strains and culturing

*C. elegans* were grown at 20 °C on nematode growth media (NGM) plates with *E. coli* OP50 as food source. The nematodes were age-synchronized by treating gravid adults with alkaline hypochlorite. The isolated eggs were rotated overnight in M9 buffer (KH_2_PO_4_ (22 mM), Na_2_HPO_4_ (42 mM), NaCl (85.5 mM)) in distilled water, autoclaved then adding MgSO_4_ (1 mM) to allow nematodes to hatch and arrest at L1 stage. The age-synchronized (L1) nematodes were placed on NGM plates ready for subsequent experiments. Wild type nematodes were sourced from the *Caenorhabditis* Genetics Centre (University of Minnesota) and the transgenic strain cop-136(P*W08E12.3/4*::GFP) was created by Mos1-mediated single copy insertion (KnudraTransgenics, USA). In addition, an extrachromosomal, multi-copy version (zsEx6(P*W08E12.3/4*::GFP)) was obtained by injecting the pPD95.75 vector containing 1115 bp of the *W08E12.3/4* promoter fused in-frame to the coding sequence of the GFP gene. A plasmid containing *rol-6* was used as co-injection marker to allow the identification of transgenic organisms.

### 2.2 Metal exposure and sample collection

Age-synchronized nematodes were exposed to Zn by adding the heavy metal in equimolar concentrations to the NGM agar and the bacterial food source (*E. coli* OP50). The cop-136 (P*W08E12.3/4*::GFP) strain was exposed to concentrations ranging from 0–800 μM Zn for 48 h for fluorescence quantitation from bulk samples. For X-ray fluorescence imaging, control (wild type) and *W08E12.3/4* RNAi worms were grown in the presence of 150 μM Zn. Developmental growth and brood size experiments were performed for control (wild type) and *W08E12.3/4* RNAi worms challenged with 200 μM Zn. The concentration ranges were selected based on publications that characterized other *C. elegans* metalloproteins.^11^,^16^

### 2.3 Quantification of gene expression using GFP alleles

GFP-tagged transgenic worms cop-136(P*W08E12.3/4*::GFP) and zsEx6(P*W08E12.3/4*::GFP) were exposed to 100, 400, 600, 800 μM Zn to evaluate changes in GFP signal intensity and also to determine the localization of expression. For single worm imaging, the transgenic worms were maintained on Zn-containing plates/OP50 from L1 to L4 stage, then imaged. L4 worms (4–8) were picked onto a glass slide carrying a drop of M9 solution to which sodium azide (2%) was added to immobilize the animals. The worms were imaged by means of an inverted fluorescence Nikon microscope, using a blue laser scanning fluorescence (*λ*_ex_ = 450–490 nm) and standard phase contrast microscopy. The images were captured using a Nikon camera (Nikon UK Ltd, Kingston upon Thames, UK) at 10× magnification and analysed by dVision software or Image J software.

For the nematode based bulk assay, transgenic P*W08E12.3/4*::GFP-tagged worms were exposed to Zn (as described above) from stage L1 to L4 stage and collected. At least three M9 washes were carried out to remove the OP50 bacteria. Octyl phenyl-polyethylene glycol (IGEPAL) was added to the tube (1 μL) to prevent the worms sticking to the pipette tips. Following a titre, 5000 worms were aliquoted into a black 96 well microplate with three repeats for each condition. The microplate was scanned at 355 nm excitation and 510 nm emission wavelength using an automated plate reader (Fluoroskan Ascent FL, ThermoFisher). Following the plate reading, the worms were imaged using a blue laser scanning fluorescence (*λ*_ex_ = 450–490 nm) and standard phase contrast microscopy in order to test the reproducibility between the bulk assay and single worm imaging.

### 2.4 X-ray fluorescence imaging (XFI)

The metal accumulation pattern in individual worms was determined by X-ray fluorescence imaging (XFI) conducted at the Stanford Synchrotron Radiation Laboratory (SSRL). Briefly, the nematodes were immobilised by adding sodium azide on a plastic slide. Zn was measured at 10 keV incident energy selected with a Si(111) double crystal monochromator. The X-ray beam was focussed at a spot size of 5 × 5 μm using a Rh-coated Kirkpatrick–Baez (KB) mirror pair (Xradia Inc). The worms were rastered continuously across the beam and Zn was monitored at each pixel by recording the intensity of the fluorescence lines using a silicon drift Vortex detector (Hitachi) equipped with Xspress3 electronics (Quantum Detectors) with a dwell time/pixel of approximately 75 ms. Calibration was performed with an X-ray fluorescence thin film standard (MicroMatter). The data were quantitatively analysed with the Micro Analysis Toolkit (Webb 2011, Palo Alto, CA, USA), which allowed the visualisation and the determination of the zinc concentration in individual nematodes.

### 2.5 Life history traits (growth and brood size)

Two toxicity assays (growth over time and brood size) were performed on wild type and *W08E(12.3–12.5)* knockdown worms in the presence or absence of Zn (200 μM). For both assays, L1 worms were plated onto control and metal supplemented NGM plates seeded with HT115 bacteria (containing either the empty RNAi vector pPD129.36 or pPD129.36 expressing *W08E(12.3–12.5*)).

For the brood size assay, after the worms reached the L4 stage, single worms were picked at L4 stage into individual wells of a 12-well plate containing NGM with or without Zn. Each worm was transferred each day at the same time onto a new well until the egg-laying period was completed. The hatched progeny from each worm was scored once the progeny reached the L2/L3 stage. The worms were maintained at 20 °C throughout the assay.

To record the change in the growth of the nematodes upon metal exposure, the flat volumetric body area was recorded from day 2 of plating until day 6 (*i.e.* from stage L2 to adult). Changes in growth were captured by taking digital pictures of each nematode every day at the same time under the same magnification of the microscope throughout the development period. The images were analysed using the Image-Pro Express software.

### 2.6 Statistics

The data derived from fluorescent quantification, XFI, life history analyses were scrutinized by one-way ANOVA testing followed by a Dunnett's multiple comparison test (Prism, Graphpad).

### 2.7 Recombinant expression of the W08E12.3 protein

*W08E12.3* was cloned, in frame, into the *NcoI* and *SalI* restriction sites of the pET29a vector system. Due to the cloning strategy, six additional nucleotides (encoding for a methionine and a glutamate) were inserted between the S-tag and the start codon. The construct sequence was confirmed by standard Sanger sequencing. The calculated theoretical mass of the recombinantly expressed protein was 17280.51 Da.

The protein was recombinantly expressed in BL21 (DE3pLysS) cells in a similar manner to that previously used for *C. elegans* metallothioneins.^16^ A single colony was picked and grown in LB broth in the presence of Kanamycin (50 ng mL^–1^) for 8 h at 37 °C. This starter culture was used to inoculate (with a ratio of 1 : 10) a larger volume of LB broth with Kanamycin. The cultures were grown to OD600 0.6 and then induced with Isopropylthiogalactoside (IPTG, 1 mM). After 30 min of induction, ZnCl_2_ (100 μM) was added to the culture and cells were grown for 5 h at 37 °C with shaking. After induction, the cells were harvested by centrifugation at 1000*g* for 20 min. The pelleted cells were frozen at –80 °C until further use.

### 2.8 Protein purification

The frozen cell pellets were resuspended in 15 mL resuspension buffer (200 mM Tris–HCl, pH 7.5; 150 mM NaCl, 1 mM DTT, 0.02% sodium azide, 0.1% TritonX) including an EDTA-free protease inhibitor cocktail (Roche). The cell suspension was processed in a cell disruptor (Constant Systems, 20.3 PSI) and the resulting mixture was centrifuged at 12 000*g* for 30 min in a floor-standing centrifuge. The supernatant was discarded and the pellet was resuspended (10 mL resuspension buffer), processed a second time in the cell disruptor, and centrifuged again. The pellet, containing inclusion bodies, was collected. The protein was extracted from inclusion bodies following the protocol by Oneda and Inouye, 1999.^24^ The inclusion bodies were dissolved in 5 mL of solubilisation buffer (0.1 M Tris HCl buffer, 6 M guanidine HCl, 0.1 M DTT, pH 7.5) and the resulting mixture was left on an orbital shaker at 4 °C overnight. The solubilised protein was added dropwise (with constant stirring) to a 100-fold volume of refolding buffer (50 mM Tris HCl, 0.1 mM Zn acetate, 1 mM DTT, 0.2 M NaCl, 10 mM CaCl_2_, 0.02% sodium azide, pH 7.5, 0.25 M l-arginine) at 4 °C. The resulting mixture contained some precipitate, which was removed by centrifugation (3000*g* for 15–20 min).

The solubilised protein from the inclusion bodies was concentrated and buffer exchanged into 20 mM NH_4_HCO_3_ by ultracentrifugation (MWCO 3 kDa, Amicon Ultra, Millipore), loaded onto a gel filtration column (FPLC 16/60 HiLoad 75 Superdex prep grade, Amersham Biosciences) connected to an FPLC system (Pharmacia Akta Purifier) and eluted using 20 mM NH_4_HCO_3_. The elution of protein fractions was monitored at 220 nm (peptide bonds and Zn–S ligand-to-metal charge transfer) and 280 nm (aromatic residues). The eluted fractions containing the pure protein were pooled and re-concentrated by ultracentrifugation (MWCO 3 kDa, Amicon Ultra, Millipore). During purification, the S-tagged protein was detected by SDS-PAGE and Western blotting employing an S-tag monoclonal primary antibody and polyclonal goat-anti-mouse immunoglobulins/HRP secondary antibody.

### 2.9 Electrospray ionisation mass spectrometry (ESI-MS)

The S-tagged W08E12.3 protein was desalted in 10 mM NH_4_HCO_3_ (pH 7.9) using a PD-10 Sephadex G-25 column (GE Healthcare) and subsequent ESI-MS was performed at acidic pH to obtain the metal-free (apo) form of the protein. The sample was made up of 40 μM protein in 10 mM NH_4_HCO_3_ and 10% methanol, with the addition of 2% formic acid. Mass spectra were recorded on a Bruker Daltonics MicroTOF fitted with an electrospray ionization source operating in positive mode. The samples were injected into the spectrometer at a flow rate of 240 μL hour^–1^. Instrument conditions were as follows: source temperature 195 °C, nebulizer 0.6 Bar, dry gas 4.5 L minute^–1^, capillary exit 100 V, skimmer1 50 V, skimer2 25.2 V, hexapole1 24.2 V, hexapole2 22.4 V, hexapole RF 450 V, transfer time 81 μs and detector TOF 2300 V. The data were recorded for 0.4–2 min over the range of 500–5000 *m*/*z*. The experimental data were smoothed and deconvoluted by means of the Bruker DataAnalysis software v. 4.0 (Bruker Daltonics).

### 2.10 Inductively coupled plasma optical emission spectroscopy (ICP-OES)

Inductively Coupled Plasma-Optical Emission Spectroscopy (ICP-OES) (Perkin-Elmer Optima 5300 DV, Model S10) was applied to determine the S and Zn content of the proteins by measuring S at 180.669 nm and 181.975 nm, and Zn at 206.200 nm and 213.857 nm. Plasma operating conditions were: argon (Ar) flow rate 13.0 L minute^–1^, auxiliary gas flow rate 0.2 L minute^–1^, nebuliser flow rate 0.8 L minute^–1^ and RF power at 1300 W. About 30 mg (1/50th of the total pellet) of the inclusion body fraction was dissolved in 600 μL of 1 M nitric acid and then diluted 10-fold for ICP-OES sample preparation. Samples from gel filtration fractions were prepared for ICP-OES in 0.1 M nitric acid at the ratio of 1 part of sample : 3 parts of ultrapure nitric acid (prepared from 72% distilled HNO_3_). The ICP-OES instrument was calibrated using S and Zn standards (1–5 ppm) prepared from 1000 ppm stocks (TraceCERT, Sigma-Aldrich). All samples were analysed using three replicate readings, with a washing time of 60 seconds for each sample. Data were analysed using the WinLab 32 software (Perkin-Elmer). The concentration of the protein was determined *via* S content from these ICP-OES measurements, based on 22 sulfur atoms from 19 cysteine and 3 methionine residues.^25^

### 2.11 pH titration

A pH titration was conducted to estimate the strength of metal binding to the S-tagged W08E12.3 protein. The protein was buffer-exchanged into 10 mM NH_4_HCO_3_ buffer (pH 7.9). pH titration of the Zn-loaded protein was monitored by UV-visible spectroscopy (Cary 50, Varian) in the range of 200–300 nm at 298 K. Respective protein samples (1.5 mL, 5–10 μM, 10 mM NH_4_HCO_3_ buffer, pH 7.9) were titrated with HCl (1–5 μL, 0.01–5 M). Each absorbance measurement was taken after 10 min of equilibration time. pH readings were taken before and after the UV spectrometric measurement.

## 3. Results and discussion

**Fig. 1:**
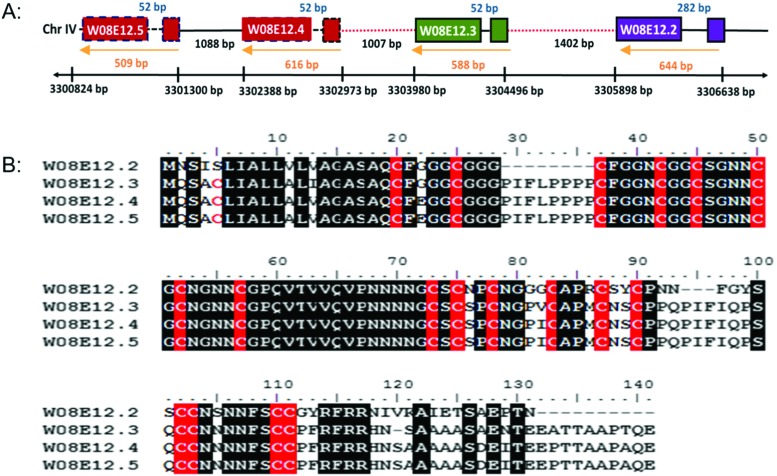
(A) Schematic diagram of a section of cosmid *W08E12* which is part of chromosome IV depicting the location of *W08E12.2*, *W08E12.3*, *W08E12.4* and *W08E12.5*. Note the coding sequences of *W08E12.4* and *W08E12.5* (red boxes), and the first 500 bp of the *W08E12.3* and *W08E12.4* promoters (red dashed lines) are identical. (B) ClustalW alignment of amino acid sequences of W08E12.2, W08E12.3, W08E12.4 and W08E12.5. Identical residues are shown in black and cysteines are highlighted in red. Note: the high level of sequence identity between W08E12.3, W08E12.4 and W08E12.5, with W08E12.2 being shorter and lacking the N terminal cysteine.

*W08E12.2*, *W08E12.3*, *W08E12.4* and *W08E12.5* are consecutively aligned on chromosome IV of *C. elegans* (; www.wormbase.org). The genomic region spanning *W08E12.2* to *W08E12.5* was cloned and sequenced, which confirmed the information (position and sequences) provided by the WormBase database (data not shown). All genes possess similar intron and exon structures and are evenly spaced out (Fig. 1A). A sequence alignment depicts the high level of sequence similarity within the respective coding regions, notably with *W08E12.4* and *W08E12.5* being 100% identical. Likewise, the promoter regions of *W08E12.3* and *W08E12.4* are identical. The predicted (*in silico* translated) protein sequences revealed that all four proteins are cysteine-rich (18–19 cysteines with a total length of 120–141aa) (Fig. 1B). A bioinformatic screen was conducted in PERL using the UVA FASTA SERVER to define how often a chain of four (or more) highly similar genes are sequentially positioned within the nematode genome. Three criteria were set, namely the identified transcripts need to be highly similar (*e* ≤ 0.0001), each chain should contain at least four genes and the gap between two consecutive transcripts should be ≤2000 bp. This identified 116 chains comprising clusters of 4–12 transcripts, 53 of which were composed of four transcripts (Table S1, ESI†). This similarity suggested that a recent duplication event may have taken place. In contrast, *W08E12.2* was found to be more different in certain parts of the coding region, possibly due to a more distant divergence. Gene duplication resulting in a tandem repeat is estimated at 60.5 and 58.8 × 10^–11^ per gene per generation in *D. melanogaster* and *C. elegans*.^26^ First, this demonstrates that gene duplication rates are comparable between flies and worms and second, emphasizes that a second duplication (resulting in a string of four genes) must be rare and perhaps underlines the functional importance of this family as the expression of tandem repeats often exceeds the sum of duplications.^27^

The gene family shares a significant sequence homology to uncharacterised genes present in other *Caenorhabditis* species, including *C. briggsae*, *C. remanei* but also with keratin associated proteins (65–75%) of *Homo sapiens* and *Mus musculus*. The hair keratin intermediate filaments are embedded in a matrix comprising keratin associated proteins, which form disulphide bonds with abundant cysteine residues of hair keratins.^28^ Cytokeratins have proven to resist Cd induced apoptosis by representing an adaptive survival mechanism.^29^ In the case of As poisoning, a temporary accumulation of the metal takes place in the soft tissue organs but the major long term accumulation takes place in keratin rich tissues such as nails, hair and skin.^30^ Cu, Hg, Ag, Cd, Pb, Cr and Al are taken up by wool keratin.^31^ Keratin protein fibres from hair, wool, horns and feather have been used to absorb heavy metals in industry for water purification and air cleaning.^32^ The homology of *W08E12.2* to *W08E12.5* with keratin associated protein confirms the possible association of the gene family with heavy metals. The JASPAR CORE database was chosen to screen for putative metal binding sites within 1000 bp of the promoter region which revealed a list of Zn-coordinating transcription factor binding sites, many of which are in common with the metallothioneins (MTs) including ELT-3, CHE-1, EOR-1 and BLMP-1. The *C. elegans* metallothioneins (MTL-1 and MTL-2) are small (75 and 63aa, respectively) cysteine-rich (19/18aa) proteins involved in essential metal homeostasis and toxic metal detoxification.^23^ Unlike most other animal genomes, the nematode does not encode for a classical MTF-1 transcription factor, and expression is believed to be modulated by ELT-2 and GATA.^13^ W08E12.2 to W08E12.5 are longer than MTLs (120–141aa), however with a comparable cysteine content (18–19 cysteines), therefore providing circumstantial evidence of a similar function (Table S2, ESI†).

**Fig. 2:**
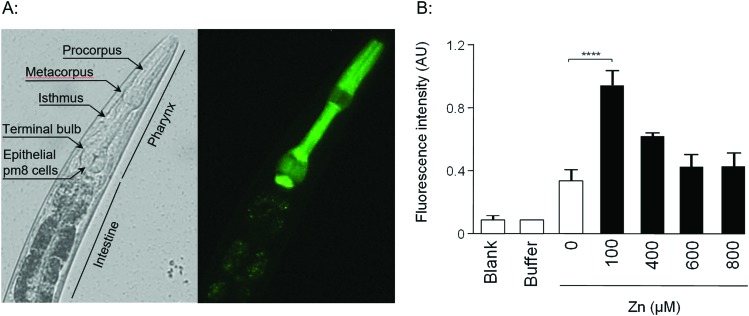
(A) A transgenic strain, cop-136(P*W08E12.3/4*::GFP), was created by Mos1-mediated single copy insertion containing the promoter sequence shared by *W08E12.3* and *W08E12.4* fused in-frame to the coding sequence of the GFP gene. Single worms were imaged by means of fluorescence microscopy (*λ*_ex_ = 450–490 nm) at 40× magnification. Note the expression is predominantly localized in the pharyngeal area of the worm (the procorpus and the isthmus, especially in the dorsal epithelial pm8 cells above the pharyngeal-intestinal valve). The image shown is an unexposed worm (*i.e.* raised in the absence of Zn supplementation). (B) Worms were also exposed to Zn (100–800 μM) from L1 to L4 larval stage (48 h) and changes in fluorescence quantified *via* a bulk assay containing 5000 worms per condition (*N* = 3 per condition) in a Fluoroskan FL plate reader (355 nm excitation and 510 nm emission wavelength). Note: exposure to 100 μM Zn significantly (***p < 0.001) increased the fluorescence of cop-136(P*W08E12.3/4*::GFP).

Given that the promoter regions of *W08E12.3* and *W08E12.4* are identical, they were used to design a P*W08E12.3/4*::GFP construct which was introduced into position ttTi5605 of chromosome II by Mos1-mediated single copy insertion yielding cop-136(P*W08E12.3/4*::GFP). In addition, an extrachromosomal, multi-copy version was created (zsEx6(P*W08E12.3/4*::GFP)) by injecting the pPD95.75 vector. Both constructs contained 1115 bp of the *W08E12.3/4* promoter fused in-frame to the coding sequence of GFP. The transgenic strain cop-136(P*W08E12.3/4*::GFP) was visualized at L4 stage using an inverted fluorescence microscope, which revealed that the expression of *W08E12.3/W08E12.4* is, at large, confined to the pharynx, and in particular pronounced in the dorsal epithelial pm8 cells present above the pharyngeal-intestinal valve (Fig. 2A), which incidentally aligns with the location of the constitutive expression (*i.e.* in the absence of metal supplementation) of *mtl-1*^13^,^14^ and supports the notion that *mtl-1* and *W08E12.3/4* may possibly function in a similar or complementary way. The location, albeit with a significantly stronger signal multi-copy expression, was confirmed with the extrachromosomal variant zsEx6(P*W08E12.3/4*::GFP) (Fig. S1, ESI†). The cop-136(P*W08E12.3/4*::GFP) transgenic strain was exposed to a concentration range (100–800 μM) of Zn from L1 to L4 stage (*i.e.* for 48 h) and changes in fluorescence were measured *via* a bulk assay using a fluorescence plate reader. Whilst the fluorescent signal of unexposed worms was statistically indistinguishable to samples chronically exposed to high concentrations of Zn (400 μM to 800 μM), a statistically significant increase in fluorescence intensity was observed in transgenic worms exposed to 100 μM Zn. (Fig. 2B), possibly due to the zinc-induced toxicity as reported by others.^33^–^35^

The location of expression remained within the pharyngeal area and thus is, at least in this context, fundamentally different to MTLs which are induced within the intestinal cells of heavy metal exposed worms.^13^,^14^ At this stage we are not able to stipulate whether the observed transcriptional induction of *W08E12.3/4* is due to a direct (*e.g. via* a zinc bound transcription factor) or indirect effect, a line of research warranting further investigations.

**Fig. 3:**
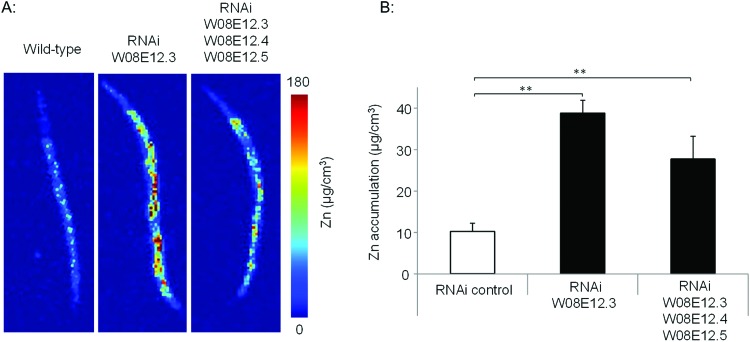
(A) Wild type and worms subjected to the knockdown (by RNAi) of specifically *W08E12.3*, or *W08E12.3 to W08E12.5* were exposed to Zn (150 μM) for 48 h and Zn accumulation was measured at 5-micron resolution by X-ray Fluorescence Imaging (XFI) at the Stanford Linear Accelerator Centre (SLAC). (B) Quantifications were conducted on 3 worms per condition and analyzed using the custom designed microprobe analysis software (SMAK).^41^ Note: knockdown of *W08E12.3* in isolation or combined (*W08E12.3 to W08E12.5)* resulted in a statistically significant (** *p* < 0.01) increase in Zn levels, predominantly in the intestinal cells.

**Fig. 4:**
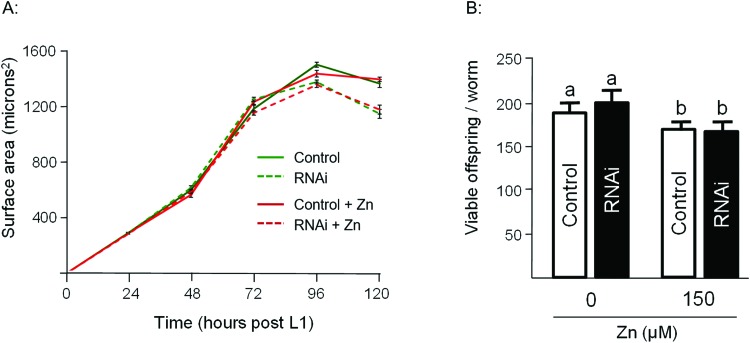
(A) Changes in developmental growth (flat volumetric area) was recorded over a 120 h post L1 stage of wild type or worms subjected to the RNAi vector of *W08E12.3*, or *W08E12.3 to W08E12.5* in the presence or absence of Zn (200 μM). Note: the marked deduction in overall surface area at the later timepoints was observed in animals subjected to RNAi but this effect was independent of Zn. (B) Total (cumulative) brood size was recorded in wild type or RNAi counterparts. Note: a statistically significant reduction in reproductive performance was observed but this was due to the exposure to Zn not the knockdown by RNAi. All values correspond to means (± SEM), *n* = 10 worms per condition for the development assay and *n* = 12 for the brood size assay. Different letters refer to a statistical difference between conditions (*p* < 0.001).

To assess the Zn-link further, the expression of *W08E12.3* to *W08E12.5* was knocked down by RNAi. Due to the sequence similarity it was possible to design a construct that was able to knock-down, in concert, *W08E12.3* to *W08E12.5* and a second construct that was specific to the knock-down of *W08E12.3.* In both cases the knock-down efficiency exceeded 50%, however due to the sequence divergence neither of the two RNAi constructs was able to knock-down *W08E12.2* (data not shown). Wild type N2 (control worms fed with HT115 bacteria containing the empty RNAi vector) and the RNAi counterparts were exposed to a dose of Zn (150 μM) for 48 h. X-ray Fluorescence Imaging (XFI) was applied to quantify the concentration of Zn in individual worms at 5 × 5 μm resolution.^36^ Upon knock-down by RNAi (using the construct targeting *W08E12.3* to *W08E12.5* and the more specific *W08E12.3)*, a statistically significant increase in Zn was observed in the gut tissue of the worms (Fig. 3A and B). The knockdown efficiency is known to decrease when multiple transcripts are targeted by RNAi^37^ thus possibly explaining the differences in metal accumulation between the two RNAi experiments. This should not distract from the fact that independent RNAi with different constructs yielded similar trends in metal accumulation, although at this point it is not possible to determine if all proteins contribute equally to the phenotype. The location of accumulation at this concentration was notably not the pharyngeal region but rather the alimentary canal, which matches the expression pattern of both metallothioneins in worms exposed to heavy metals. The metal accumulation pattern is therefore comparable to other proteins which are involved in the homeostasis/detoxification of metals, despite of the fact that the expression of *W08E12.3/4* remains within the pharyngeal region.^14^ RNAi knockdown experiments were also applied to assess whether the silencing of the genes exerts an effect on growth and reproductive output, two sensitive phenotypic endpoints previously linked to Zn exposure in worms.^38^ The growth (flat volumetric surface area) of wild type and RNAi knock-down worms was comparable for the first 72 h (post L1 stage). Thereafter, growth retardation was observed in worms subjected to the knock-down of *W08E12.3* to *W08E12.5* in comparison to wild type worms fed the (empty) RNAi vector. The observed inhibition of growth was however independent of Zn exposure (Fig. 4A). In contrast, Zn exposure resulted in a significant reduction in reproductive performance which was not further affected by the knock-down of *W08E12.3*–*W08E12.5* (Fig. 4B). No significant changes in lethality were observed in worms subjected to the knockdown by RNAi (data not shown).

**Fig. 5:**
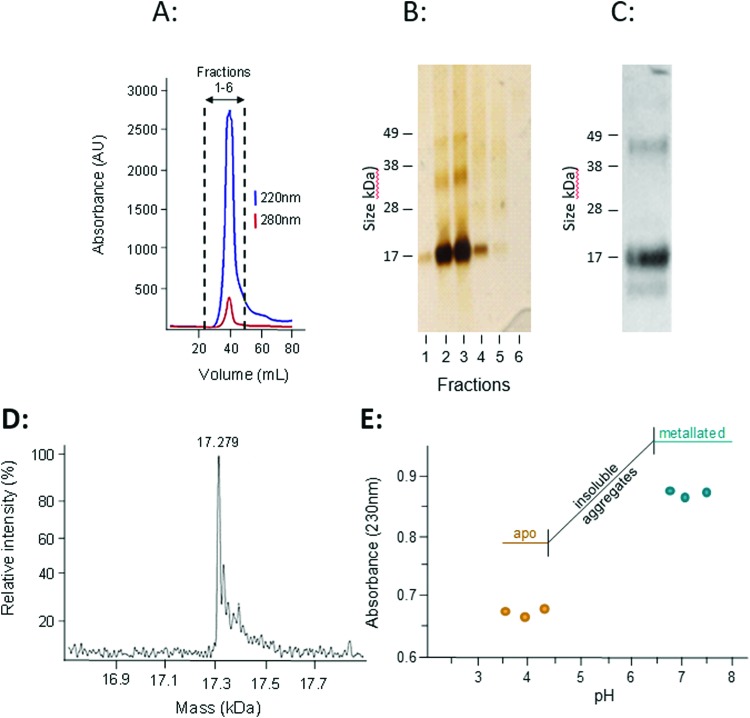
(A) An S-tagged version of W08E12.3 was recombinantly expressed in BL21 DE3(pLysS) and purified by gel filtration chromatography (HiLoad 16/60 Superdex 75, Amersham Biosciences), with absorbance of peptide bonds (220 nm) and aromatic side-chains (280 nm) monitored. (B) The fractions were separated by means of SDS-PAGE and proteins visualised by silver staining. (C) The major peak was pooled and analysed by SDS-PAGE using an anti S-tag antibody. Note the presence of an intense band representing the monomer but also a band at higher molecular weight (likely a dimerized form). (D) The accurate mass of the purified protein, as determined by ESI-MS, was 17279 Da which is very close to the calculated theoretical mass of 17280.5 Da. (E) To estimate the strength of Zn binding, a pH titration (with 1 M HCl) was conducted by measuring the absorbance of the purified protein at 230 nm. Note: the recombinantly expressed W08E12.3 aggregated between pH 6.4 and 4.3, but re-dissolved at lower pH. The decreased absorbance suggests that Zn was released from the protein at lower pH.

The presence of transcription factor binding sequences related zinc homeostasis in the promoter regions of the *W08E12.2*–*W08E12.5* genes, together with the cysteine-rich nature of the translated proteins, raises the question whether the proteins possess zinc-binding ability. Thus, *W08E12.3* was selected for further characterisation at the protein level. Protein was generated by means of recombinant expression in *E. coli*, with culture media being supplemented with 100 μM Zn(ii). Analysis of the soluble and insoluble fractions of the cell lysates revealed that the W08E12.3 protein was predominantly present in the insoluble fraction, *i.e.* in inclusion bodies. Attempts to isolate protein expressed without any tags by classical chromatography were unsuccessful (data not shown), but the S-tagged version of W08E12.3 was found to be more stable and easier to purify. However, the subsequent cleavage of the S-tag with thrombin resulted in significant protein loss, a problem that was also encountered when S-tagged protein was captured *via* S-protein affinity chromatography (data not shown). Given that the length of the 15aa S-tag contributes only 10% of the recombinant protein and has no significant metal-binding capacity, it was assumed not to interfere with protein folding.^39^ The S-tag containing W08E12.3 protein extracted from the inclusion bodies was refolded by dilution under reducing conditions in the presence of Zn (100 μM) at pH 8 and purified by gel-filtration chromatography. Due to the cysteine content, all purification steps were conducted in the presence of reducing agents (DTT or TCEP) and Zn(ii) to minimize oxidation. Protein-containing chromatography fractions were assessed by means of denaturing SDS-PAGE followed by western blotting using an S-tag antibody. This facilitated the identification of a prominent band that corresponded to the size of the monomeric W08E12.3 S-tagged protein, but also a small amount of dimer (Fig. 5A–C). Dimerization was not observed in subsequent MS experimentation, thus it was not possible to conclude whether the metallated protein was able to dimerize. The addition of Zn to the culture medium enhanced the protein yield, providing circumstantial evidence that Zn may interact with (and stabilize) the protein (Fig. S3, ESI†).

The subsequent analysis by mass spectrometry at pH 3.8 determined the mass of the metal-free apo-protein to be 17 279 Da (Fig. 5D), which is in reasonable agreement with the theoretical mass (17280.5 Da).

Inductively Coupled Plasma Optical Emission Spectrometry (ICP-OES) was used to determine whether the protein expressed in *E. coli* was likely to bind Zn, and whether the purified protein had Zn-binding ability. The Zn and S contents of unprocessed inclusion bodies were determined after dissolution in nitric acid and assuming that all S originated from W08E12.3 protein the metal : protein ratio was calculated to be 5 : 1. A higher metal : protein ratio of 6.5 : 1 was observed in the purified S-tagged W08E12.3 protein (Table 1), which is similar to MTL-1 (7 Zn) and MTL-2 (6 Zn).^15^ This suggests that the protein is, in principle, capable of sequestering Zn, however is deposited in inclusion bodies of *E. coli* likely as a mis-folded protein.

**Table 1:** The protein and Zn concentrations derived by ICP-OES and the resulting protein : metal ratio of the protein pool isolated from the *E. coli* inclusion bodies compared to the purified S-tagged W08E12.3 fractions

Sample name	Protein concentration (μM)	Zn concentration (μM)	Protein : Zn ratio
S-tagged W08E12.3 within the inclusion bodies	48	245	1 : 5
Gel filtration column purified S-tagged W0812.3	35	244	1 : 6.5

The zinc-binding strength of the metallated W08E12.3 protein was probed *via* a pH titration monitored by UV absorption spectroscopy, an approach widely used to characterise metallothioneins.^40^ The Zn–S Ligand-to-Metal-Charge-Transfer (LMCT) band was monitored at 230 nm (Fig. 5E). The Zn-bound protein was titrated with acid (HCl at different concentrations) across a pH range of 7.5 to 3.5; however the W08E12.3 protein became insoluble and aggregated between pH 6.4 and 4.3 (which coincides with its theoretical isoelectric point, pI = 5.55) and precluded the determination of a pH of half-displacement. However, the W08E12.3 protein re-dissolved at lower pH; the reduced absorption value at 230 nm confirmed that Zn loss was, in principle, pH dependent as expected. The likely complete demetallation by pH 4.3 suggest that if a pH of half-displacement could be determined, it would be at least one pH unit higher, *i.e.* significantly higher than those for most metallothioneins.^21^ Of course the above evidence does not rule out the possibility that other metals may be able to bind to these proteins and function in a zinc-independent manner. For example, using bioinformatics tools *via* the MyHits database Motif Scan revealed that W08E12.3 possesses a 2Fe–2S ferredoxin-type iron–sulfur binding region in the sequence. Thus, exposing the transgenic nematodes to different iron sources will unravel if this ferredoxin-type iron–sulfur binding region could be responsible for the metal ion-dependent transcriptional activation of *W08E12.3*. Preliminary data suggest that this is the case: supplementation of the media with 30–200 μM iron(ii) induced the fluorescence in transgenic animals by 36–46% (24 h exposure) or 41–63% respectively (48 h exposure) (Fig. S2, ESI†).

## 4. Conclusions

The data obtained by the *in silico* metallomics screen, the *in vivo* gene expression analysis, targeted silencing by RNAi with follow-up studies investigating changes in metal accumulation and phenotypic endpoints as well as protein level studies have led to the identification and initial characterization of a closely related family of four metalloproteins, named W08E12.2, W08E12.3, W08E12.4 and W08E12.5 according to their position on a cosmid. The transcripts are metal-responsive and the resultant W08E12.3 protein is capable of binding Zn. The W08E12.2 to W08E12.5 family share some functional features of metallothioneins, namely several transcription factor binding sites within the promoters, the location of expression and metal responsive properties, changes in Zn metabolism following silencing by RNAi, and the capacity of binding and the proton-induced release of Zn from the protein. Taken together, this study has uncovered a new family of metalloproteins which may function in metal sensing, metal homeostasis and/or metal detoxification, not dissimilar to metallothioneins.

## Conflicts of interest

The authors declare that there are no conflicts of interest.

## Supplementary Material

Supplementary information
