# Supplementary material for: Molecular genetic and biochemical characterization of a putative family of zinc metalloproteins in Caenorhabditis elegans
Source: Metallomics. 2018 Nov 16;10(12):1814–23. doi: 10.1039/c8mt00169c (PMC6336089; doi:10.1039/c8mt00169c)

Electronic Supplementary Material (ESI) for Metallomics.
This journal is © The Royal Society of Chemistry 2018

Supplementary Table 1: Identified gene clusters in each chromosome of the *C.elegans* genome. and their frequency of distribution within each chromosome. A PERL-based bioinformatic screen was conducted to quantify the likelihood that four (or more) highly similar genes are sequentially positioned within the genome of *C.elegans*. Any chain of transcripts had to be highly similar (e ≤ 0.0001), contain at least four genes and the gap between two consecutive transcripts should not exceed 2000 bp. Note only few chains of genes met the above criteria suggesting that this is a relatively rare event within the worm genome.

Chr	Length (Mb)	Protein coding genes	Chain of 4 genes	Chain of 5 genes	Chain of 6 genes	Chain of 7 genes	Chain of 8 genes	Chain of 9 genes	Chain of 10 genes	Chain of 12 genes	Number of chains	Frequency (chains/ protein coding genes)
1	15.08	2920	1	1	1	1	0	0	0	0	4	0.0013
2	15.28	3552	24	12	6	3	0	0	1	0	46	0.0129
3	13.78	2699	5	2	0	1	0	0	0	0	8	0.0030
4	17.49	3348	8	7	1	2	2	0	0	1	21	0.0062
5	20.92	5149	12	11	2	1	0	1	0	0	27	0.0052
X	17.72	2844	3	4	2	1	0	0	0	0	10	0.0035
Total	100.27	20512	53	37	12	9	2	1	1	1	116	0.0056

Supplementary Table 2: Identified transcription factor binding sites positioned within the first 1000 bp upstream of the promoters of *W08E12.2, W08E12.3 W08E12.4, W08E12.5*, *mtl-1*, and *mtl-2*. The screen was conducted using the JASPAR CORE database based on a curated, non-redundant set of profiles, derived from published collections of experimentally defined transcription factor binding sites for eukaryotes. Note: The screen identified several common Zn-coordinating transcription factor binding sites such as ELT-3, CHE-1, EOR-1 and BLMP-1, suggesting a possible (functional) linkage between the two families.

Transcription factor	*W08E12.2*	*W08E12.3*	*W08E12.4*	*W08E12.5*	*mtl-1*	*mtl-2*
CHE-1	21	16	16	18	13	19
ELT-3	10	5	5	3	9	6
BLMP-1	15	15	15	4	42	23
MAB-3	1	1	1	0	1	0
DAF-12	2	0	0	3	2	0
EOR-1	0	0	0	1	8	2

Supplementary Figure 1: Comparison of fluorescence signal between (A) the integrated single copy transgene cop-136(P*W08E12.3/4*::GFP) and (B) the extrachromosomal multicopy version zsEx6(P*W08E12.3/4*::GFP). Note: The GFP signal is observed at the same location within the pharynx of either strain, thus serving as an independent validation of the expression. Due to the multi-copy (however less stable) nature of the extrachromosomal array, the signal intensity is amplified.

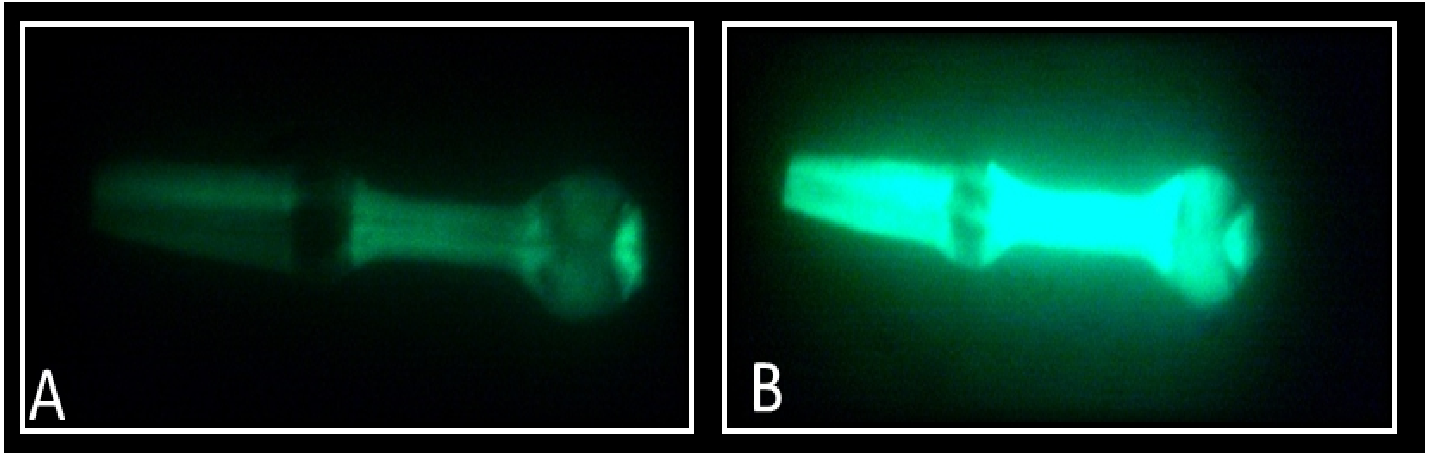

Supplementary Figure 2: Change in fluorescence intensity in the nematode *pW08E12.3::GFP* following an exposure to iron(II). All nematodes were age synchronized and imaged at L4 stage using an inverted fluorescence microscope with a GFP filter. (A) Fluorescence intensity after an exposure to iron(II) for 24 h or (B) after 48 hour, *(n=10), error bars =SD*, *** *p<0.001.*

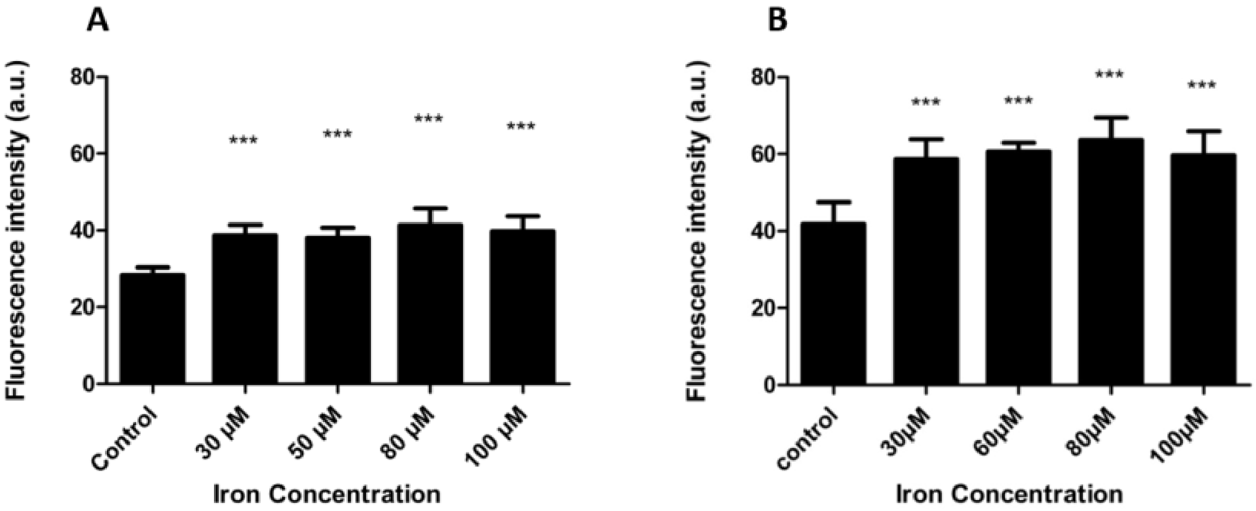

Supplementary Figure 3: Western blot with an anti S-tag antibody of the W08E12.3 protein generated via the S-tag system. The cultures were sonicated, and the samples boiled in Laemmli's buffer, then loaded on to an SDS-PAGE gel. Lane 1: supernatant obtained from culture supplemented with 100 µM $ZnCl_2$; Lane 2: supernatant of culture (without added Zn), Lane 3: pellet obtained from the culture supplemented with 100 µM $ZnCl_2$ following an extraction using urea, Lane 4: pellet obtained from the culture (without additional Zn) followed by an extraction using urea. Samples were normalized to the same concentration prior to loading.

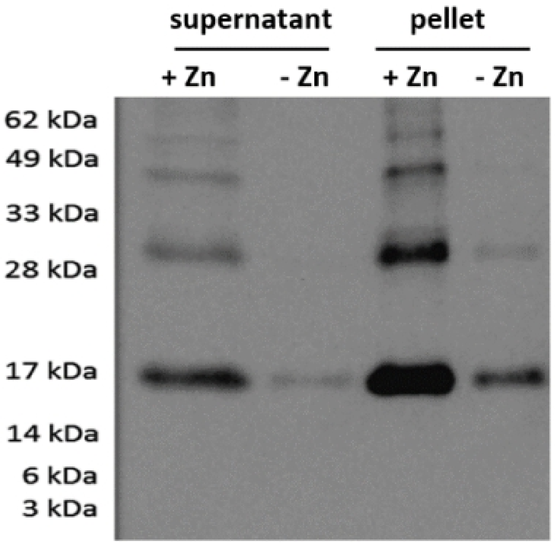

Supplement: Supplementary file 1 [file MT-010-C8MT00169C-s001.pdf]
